# Hemodynamic responses in human multisensory and auditory association cortex to purely visual stimulation

**DOI:** 10.1186/1471-2202-8-14

**Published:** 2007-02-06

**Authors:** Martin Meyer, Simon Baumann, Sarah Marchina, Lutz Jancke

**Affiliations:** 1 Institute of Neuroradiology, University Hospital of Zurich, Switzerland; 2 Department of Neuropsychology, University of Zurich, Switzerland; 3 School of Neurology, Neurobiology and Psychiatry, Newcastle University, UK; 4 School of Psychology, Brain & Behaviour, Newcastle University, UK

## Abstract

**Background:**

Recent findings of a tight coupling between visual and auditory association cortices during multisensory perception in monkeys and humans raise the question whether consistent paired presentation of simple visual and auditory stimuli prompts conditioned responses in unimodal auditory regions or multimodal association cortex once visual stimuli are presented in isolation in a post-conditioning run. To address this issue fifteen healthy participants partook in a "silent" sparse temporal event-related fMRI study. In the first (visual control) habituation phase they were presented with briefly red flashing visual stimuli. In the second (auditory control) habituation phase they heard brief telephone ringing. In the third (conditioning) phase we coincidently presented the visual stimulus (CS) paired with the auditory stimulus (UCS). In the fourth phase participants either viewed flashes paired with the auditory stimulus (maintenance, CS-) or viewed the visual stimulus in isolation (extinction, CS+) according to a 5:10 partial reinforcement schedule. The participants had no other task than attending to the stimuli and indicating the end of each trial by pressing a button.

**Results:**

During unpaired visual presentations (preceding and following the paired presentation) we observed significant brain responses beyond primary visual cortex in the bilateral posterior auditory association cortex (planum temporale, planum parietale) and in the right superior temporal sulcus whereas the primary auditory regions were not involved. By contrast, the activity in auditory core regions was markedly larger when participants were presented with auditory stimuli.

**Conclusion:**

These results demonstrate involvement of multisensory and auditory association areas in perception of unimodal visual stimulation which may reflect the instantaneous forming of multisensory associations and cannot be attributed to *sensation *of an auditory event. More importantly, we are able to show that brain responses in multisensory cortices do not necessarily emerge from associative learning but even occur spontaneously to simple visual stimulation.

## 1 Background

Associative learning and adaptation can be regarded as one of the most fundamental behavioural functions in both humans and animals. The processing of external and internal sensations is an important prerequisite for learning. Feeling the painful heat while viewing and touching a hot plate helps an organism learn about a potential danger. In this example, the combination of multiple visual and somatosensory sensations enables an organism to establish an association between an object, the hot plate, and a painful action and thus helps in acquiring an appropriate behaviour. But even paired sensations that are not unpleasant clearly demonstrate that multisensory learning may establish a strong relationship between two events. On seeing lightning individuals immediately anticipate hearing thunder based on previous experience. Thus, inputs from the different sensory modalities are combined to form a single integrated experience of the world [1,2]. Multisensory sensations and integrations are therefore enormously important and advantageous tools in an organism's repertoire to effectively learn how to act properly and how to avoid deleterious experiences. For example, recent animal research has provided compelling evidence of visual and somatosensory input into putatively unisensory regions at the secondary/tertiary levels of the auditory hierarchy [3,4]. The existence of at least three types of heteromodal connections linking unimodal sensory (visual, auditory, and somatosensory) cortices in the monkey brain has been recently demonstrated by a study using retrograde tracers [5]. At the neurofunctional level recent electrophysiological studies using intracranial recordings from humans and animals have shown direct visual and somatosensory input to the caudomedial belt area of auditory association cortex [6,7].

Relative to the knowledge obtained from animal research, to date little is known about the neural underpinnings of multisensory learning in the human brain. So far, a paucity of brain imaging studies has demonstrated the existence of functional coupling and structural connections across modalities which are supposed to constitute basic mechanisms of learning [8,9]. At least with regard to cortical networks there is evidence indicating that primary and associative sensory regions preferentially bind together to enable multisensory learning. Using functional magnetic resonance imaging (fMRI), Foxe and colleagues demonstrated that auditory and somatosensory inputs converge in a subregion of human auditory cortex along the superior temporal gyrus (STG) [10]. Multisensory processing has also been the subject of imaging studies on visual memory retrieval. For example, in an fMRI study Nyberg and colleagues observed that visual retrieval of auditory presented words activates the core auditory cortex [11]. Interestingly, recent investigations on auditory imagery evoked by visual cues have also shown that mental imagery of complex auditory percepts brings on activation increases in secondary auditory fields [12-14]. Results of another fMRI-study indicated that mentally recalling learned sounds yields enhanced activation in human auditory association cortex [15]. By virtue of these meager findings it has become a current matter of research whether highly associative unimodal stimuli are more likely to activate primary sensory regions during crossmodal learning or whether these unimodal stimuli recruit polysensory and auditory association cortices to establish learned representations.

All these aforementioned studies have in common that they encourage participants to embark on a controlled top-down strategy. However, it has also been shown that automatic bottom-up processing may trigger audio-visual intertwining. For example, one fMRI-study uncovered responses in the visual cortex to presentation of sounds in isolation following a learning period in which a visual stimulus was consistently paired with an audible tone [16]. In this study McIntosh and colleagues demonstrated multisensory interactions characterized in human subjects as they learned that an auditory stimulus signals a visual event.

### The present study

Thus, we set up a study involving human participants which is similar to the cited experiment by McIntosh and colleagues, but addressed the question whether visual stimuli may induce activity in polysensory and auditory association cortices or auditory core regions after they had been presented in combination with sounds. We used a conditioning paradigm which taps the simplest form of associative learning by establishing a short-term relationship between two events even when these events are affectively neutral and have no relevance for the organism that undergoes conditioning. This form of learning occurs when a previously neutral stimulus (CS) is temporally paired with another unconditioned stimulus (UCS) that evokes a physiological and/or behavioural response (UCR). After a phase of consistent temporally paired stimulation (short-delay conditioning) it suffices to present the formerly neutral stimulus (now CS+) to observe the response initially elicited by the UCS. A spontaneous association is even formed when the CS and UCS do not have any natural linkage or when CS is presented outside of awareness [17]. Unlike previous imaging studies which applied aversive auditory and tactile stimuli [18,19], we applied non-aversive simple sensory stimuli to avoid confounding with emotional processes.

Our specific hypotheses were as follows:

#### Principal hypothesis

Based on previous findings from neuroimaging studies on bottom-up multisensory processing [16] we assume an involvement of polysensory and auditory association areas triggered by the presentation of visual stimuli in isolation which have precedingly been paired with auditory stimuli.

#### Additional hypothesis

According to the observations of Schroeder and colleagues who consider the auditory association cortex in the posterior Sylvian fissure and the superior temporal sulcus that corresponds to the superior temporal polysensory area in the macaque neocortex, as essential candidate regions for audio-visual processing [3], we predict an involvement of these areas in the context of the present crossmodal paradigm.

#### Additional hypothesis

Based on the results of an aforementioned PET-study on classical conditioning by Hugdahl and colleagues we conjectured that inferior frontal regions may also play a role in associative learning as the inferior frontal cortex has been described as an additional supramodal resource which supports the establishment of functional relationships in crossmodal conditioning [19].

## 2 Results

**Figure 1:**
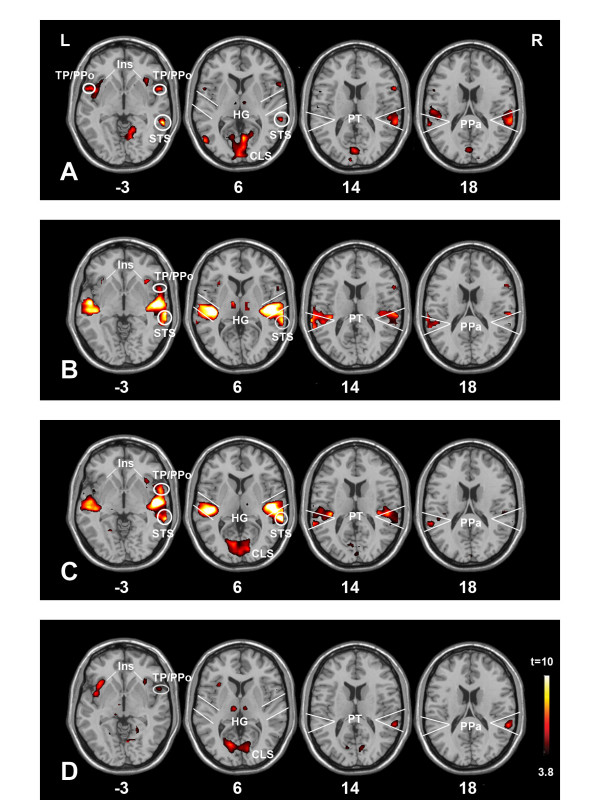
Functional brain responses collected during the four experimental phases are depicted. The brain scans show consistently stronger functional activation for stimuli conditions relative to silent control obtained from the second out three volumes. All functional contrasts are thresholded at *T *= 3.79, *p *≤ 0.001 (uncorrected *α*-level, *k *≥ 10) and superimposed on transverse and sagittal slices of the MNI-T1-weighted standard brain. Tables 1-4 list peak activations (T-values) of distinct activation clusters and anatomical areas. [A] Visual habituation, [B] Auditory habituation, [C] Conditioning phase, [D] Test phase (extinction).

Figure 1 and tables 1, 2, 3, 4 display the main fMRI results. All main contrasts reported here are derived from the comparison between the experimental phases vs. silent control (null events) to farthom the entire ensemble of involved brain areas.

### First phase (visual control)

Figure 1A and table 1 illustrate that perception of visual stimuli was associated with activity in several cortical areas normally attributed to visual processing, namely the calcarine sulcus (CLS), the cuneus (Cun), and the left temporo-occipital lobe (V4). We also noticed robust activation of the bilateral supramarginal gyrus (SMG) which overarches the planum parietale (PPa), of the posterior auditory association cortex including the planum temporale (PT) and of the right posterior portion of the superior temporal sulcus (STS). Furthermore, we observed bilateral activity situated rostrally and dorsally to auditory core regions, namely in the opercular part of the inferior frontal gyrus (IFG(op)), in the anterior insula, in the temporal pole, and in the Rolandic operculum. Finally, the analysis revealed recruitment of subcortical thalamic and basal ganglia (Putamen) sites.

### Second phase (auditory control)

Figure 1B and table 2 visualize that hearing simple telephone ringing elicits considerable hemodynamic respones in primary and secondary auditory fields stretching along the entire ventral bank of the perisylvian region including the supratemporal plane as well as the lateral STG. Furthermore, we uncovered small patches of activity in left and right anterior insulae as well as in the thalamic area.

### Conditioning phase (paired vision (CS) and audition (UCS))

Figure 1C and table 3 depict brain responses while participants were presented with paired visual and auditory stimuli. The analysis identified significantly stimulated regions in the superior temporal region and in the anterior insulae bilaterally, in the calcarine sulcus, in the cuneus and in the inferior colliculus.

### Maintenance (paired vision (CS-) and audition (UCS))

We do not explicitly report activation evoked by the maintenance condition as it turned out to involve the same regions as the preceding conditioning phase.

### Test phase or extinction (only vision (CS+))

Figure 1D and table 4 visualize areas that were active while participants only viewed stimuli after conditioning had occurred. Besides responses in the visual cortex (CLS), in the right lingual gyrus, and in the right cuneus/precuneus we also identified right lateralized activity in auditory association regions of the posterior Sylvian fissure partly encroaching onto the PT and the adjacent PPa. In the right hemisphere we also observed an activation cluster which covered the IFG(op) and the anterior temporal plane. Furthermore we found bilateral responses in the anterior insula. Please note that "maintenance" trials were presented randomly interspersed and alternating with "extinction" trials within the same run.

#### Post-hoc analysis

**Figure 2:**
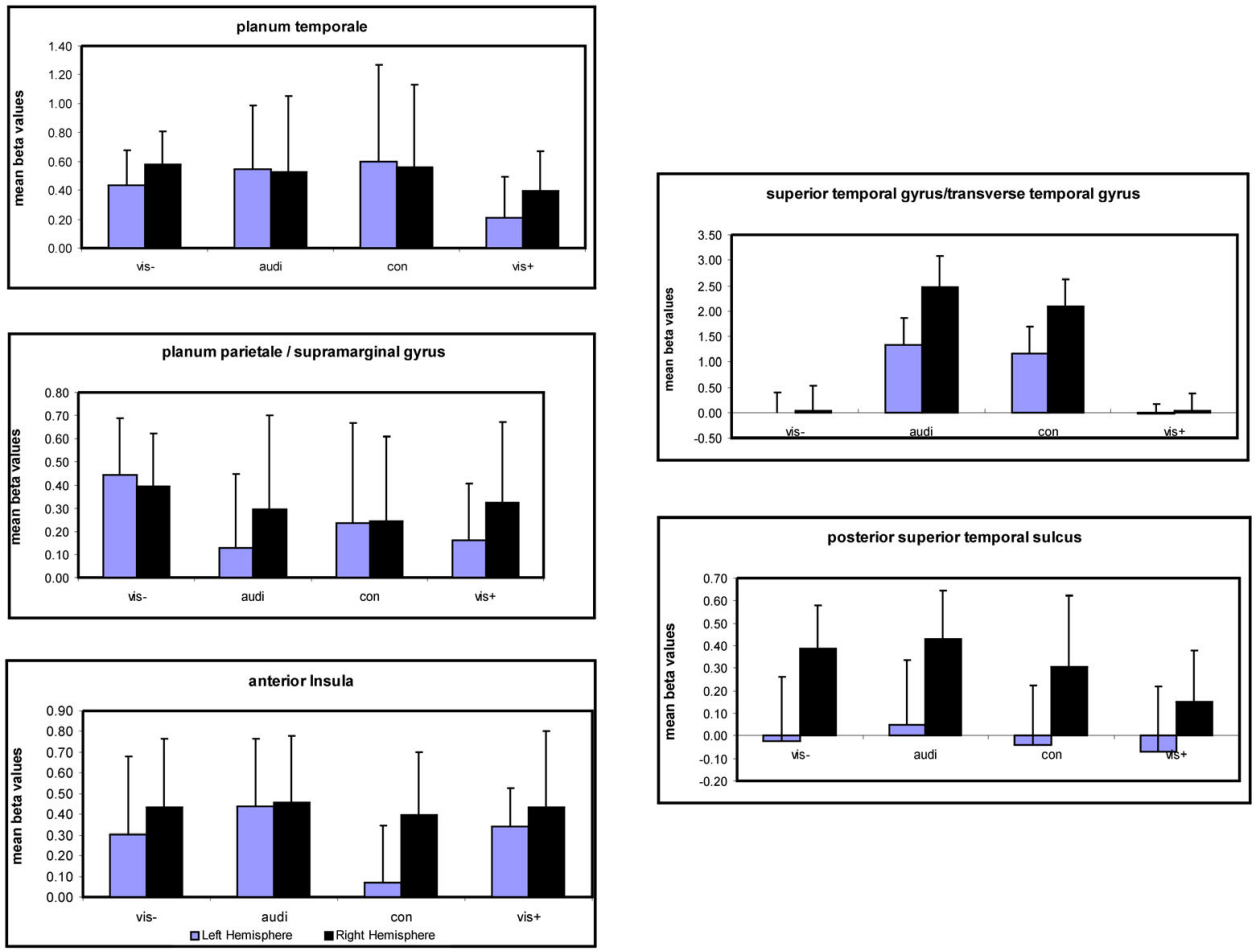
Results of spherical ROI analysis. Mean *β*-values collected from five bilateral distinct regions in the fronto-temporo-parietal cortex. Error bars refer to the standard deviation.

Figure 2 and table 5 show that mean *β*-values in frontal, temporal and inferior parietal sites differ intra- and interregionally as a function of the experimental run and hemisphere.

We subjected *β*-values to global 4 × 5 × 2 ANOVA with factors *phase *× *ROI *× *hemisphere *that revealed a main effect of *phase *(*F*_3,12 _= 31.05, *P *< .0001), a main effect of *ROI *(*F*_4,11 _= 21.40, *P *< .0001), and a main effect of *hemisphere *(*F*_1,14 _= 49.26, *P *< .0001). The latter main effect points to a general superiority of right hemisphere ROIs in the context of the present study. Furthermore the ANOVA evinced interactions of *phase *× *ROI *(*F*_12,3 _= 34.5, *P *< .0001), *phase *× *hemi *(*F*_1,14 _= 7.67, *P *< .0001), *hemi *× *ROI *(*F*_3,12 _= 16.11, *P *< .0001), and *phase *× *ROI *× *hemi *(*F*_12,3 _= 9.48, *P *< .0001). Based on the results of the global ANOVA we performed a separate (4 × 2) ANOVAs with factors *phase *× *hemisphere *for each ROI. Table 5 shows the results of these ANOVAs which indicate that the pattern of BOLD responses differed considerably within the distinct ROIs as a function of experimental phase, that is the absence or presence of stimulation respectively.

First, these analyses also demonstrate that the core auditory region (HG/STG) is only involved when participants listened to auditory stimuli. By contrast, the statistical comparison evidence that multisensory and auditory association regions (PT, PPa, post. STS) are without exception more strongly activated during the first visual phase prior to conditioning relative to the last visual phase that followed the conditioning phase (main effect of *phase *(*F*_1,14 _= 9.69, *P *< .01)) with the right hemisphere being more strongly involved (main effect of *hemisphere *(*F*_1,14 _= 12.26, *P *< .005)).

In contrast to the auditory core region, posterior auditory association areas (PT, PPa) show no significant phase effect indicating that we observed surprisingly strong crossmodal activation.

## 3 Discussion

The current study was designed to demonstrate that isolated presentation of visual stimuli which have been paired with auditory stimuli prior to isolated presentation activates regions associated with auditory perception. Interestingly, we noticed also functional responses in multisensory and auditory association cortex to visual stimuli that were significantly stronger during the first visual phase, that is *before *the paired presentation of flashes and sounds relative to the last phase which was assumed to show strongest multisensory responses. Even though this main finding of the present study is surprising it can be given a plausible explanation. Apparently the stimuli we used in this conditioning experiment turned out to be behaviourally more relevant than we were aware of when designing the study. A number of our participants reported that they experienced the red flash as "alarming" and "startling". A similar experience was reported by subjects when we debriefed them as to how they experienced the telephone ringing. Thus, our major finding suggests that even the pure presentation of visual and, to some extent, auditory stimuli established rapid visual-auditory associations supported by multisensory and auditory association cortices.

In support of our interpretation we refer to recent observations made in human and animal studies that investigated to what extent motor, visual, and somatosensory stimuli induce responses in multisensory and auditory associative regions. Interestingly, these studies also report involvement of the same multisensory areas that we found in the present study, namely the inferior parietal lobe (IPL), the posterior auditory association cortex, and the right superior temporal sulcus [20,10,21,6]. After the discussion of the unequivocally perceptually related responses in the primary and secondary auditory cortices we will broach the issue of multisensory associations in the context of our main finding in more detail.

### Primary and secondary auditory and visual cortex

As the statistical maps and the ROI analyses demonstrate, listening to auditory stimuli in isolation or paired with visual stimuli results in a salient fMRI activation in the core auditory cortex bilaterally. Interestingly, these regions are not engaged during the presentation of visual stimuli in isolation. This finding can be taken as strong evidence for the view that primary auditory cortices on the bilateral supratemporal plane and on the lateral convexity of the superior temporal gyrus are not sensitive to multisensory input coming from visual or somatosensory territories. Unlike the multisensory and auditory association cortices we discuss below, the core auditory fields are driven exclusively by explicit auditory input. The involvement of primary visual cortex was only observed when visual stimuli were presented in isolation or combined with auditory stimuli. Here again, it appears that primary regions of one domain (visual) are not amenable to crossmodal perception. Taken together, these findings do not evidence the existence of direct connections between primary and auditory cortices. Should they exist we assume that they are not sufficient to evoke a BOLD dependent fMRI response. At any rate, connectivity to auditory association cortices (PT/PPa) seems to be much stronger. With respect to activation of the left V4 region (MTG/MOG) unveiled in the first run only we suggest that decoding of the stimulus' colour led to its activity as this region has been attributed to colour perception [22].

### Posterior Sylvian fissure

The results obtained by the ROI analysis reveal robust involvement of the posterior Sylvian fissure accommodating the PT and parts of the inferior parietal lobe (IPL), namely the planum parietale and the supramarginal gyrus during the different experimental phases. While the ROI analyses show that responses in the IPL during the initial visual phase are significantly stronger relative to the other experimental phases, the activation in the PT was initially stronger only when compared to the last visual run, but not relative to the conditions during which auditory stimuli were also presented. Evidently, this finding may indicate that the PT is to some extent also recruited in the perception of the presented auditory stimuli. We will first discuss the find-ing of IPL activation, followed by a discussion of the PT involvement.

Based on the present knowledge of connectivity and function of the IPL obtained from animal research and human fMRI studies we predicted an involvement of this region in our conditioning paradigm. Akin to the PT and the right STS the initial visual phase of the study brought on a considerable signal increase in the IPL. This region has been described as part of the macaque dorsal auditory stream originating from the caudal part of the STG and projecting to the parietal cortex which preferentially responds to auditory spatial information [23]. Numerous functional imaging and clinical studies in humans support this observation obtained from animal research [24-27]. Evidently, it has been demonstrated that the human IPL which harbors the SMG/PPa is involved in associative auditory source localization but also in the discrimination of formant structure of male and female voices [28]. Another function associated with this area has recently been described by Gaab et al. [29] who showed that SMG bilaterally (but mainly on the left) subserves working memory for tonal information and should therefore be considered a region that is essential for higher auditory functions. An involvement of the IPL and the adjacent parietal operculum in auditory imagery of music has also recently been reported by an fMRI study which tested expressive and perceptive aspects of crossmodal auditory-motor functions in professional pianists [20]. Thus, we reason that rapidly occuring multisensory responses to visually presented cues are likely to account for our result.

The PT subserves a variety of genuine auditory functions, i.e. processing of auditory spectrotemporal information [30-32], temporal integration of sequential auditory events [33], neural representation of pitch information available in tonal and nontonal languages [34], the discrimination of novel from known sounds [35], analysis of changes in spectral envelope and fundamental frequency [36] but also auditory imagery of linguistic and non-linguistic information [12,13,37]. In particular, the latter study is of interest as it demonstrates enhanced activity in the left PT when individuals had to attend to pure visually presented speech gestures and thus supports the view of the planum temporale as multisensory area. Neuroplastic changes in the PT have also been demonstrated by studies that investigated the comprehension of sign language. These studies observed that in congenitally deaf individuals the PT responds to visually presented linguistic information [38-40]. Anatomical research also provides evidence for a direct interhemispheric input from the right extrastriate visual cortex to left auditory regions, in particular to the planum temporale [41]. However, to the best of our knowledge we are not aware of any anatomical study that reports direct homo- or heterotopic connections from primary, or secondary visual cortex to the multisensory regions in the posterior Sylvian fissure so that we can only hypothesize the potential existence of neural connections that enable a rapid tight coupling of visual and auditory regions. In the context of the present study we conclude that activity in the planum temporale conjointly and immediately occurred to support multisensory perception as this region is involved in the perception of both pure visual and auditory stimuli.

### Superior temporal sulcus

As apparent from figure 1 and from figure 2 we observed an involvement of the right posterior STS regardless of modality. This finding came as no surprise as the human STS has been described as a heteromodal area that corresponds to the polysensory STP area in the macaque cortex [3]. Due to its connections to the auditory cortex and to temporo-occitpital association areas ("Plis de passage" [42] this associative cortex is presumed to bind information coming from unimodal sensory areas and thus may help form crossmodal associations [43]. According to a recent fMRI study the STS should be considered a region where auditory and visual information about objects is integrated [44]; it has also been noted to play a cardinal role in audiovisual speech perception [30,45,46]. While the latter studies associate the left hemisphere STS with crossmodal representations during audiovisual speech perception, our present finding of dominant activation in the right posterior STS (approximately 40 mm posterior from the anterior commissure) calls for another interpretation. The majority of these studies associate the posterior right STS with socially and behaviourally relevant visual cues, namely biological motion and static images of the face and the body [47]. Wright and colleagues [48] localized stronger responses to paired audiovisual stimuli (movie of animated character moving her mouth) relative to isolated presentation of visual and auditory stimuli in a portion of the right posterior STS (40–55 mm posterior from the AC) that overlaps with the STS cluster we observed in the present study. Even though the auditory and visual stimuli we presented to our participants (paired or isolated) were less complex than the animated characters used by Wright and colleagues, they were of apparent behavioural relevance. Thus, we infer from our results that the presentation of auditory and visual stimulation in our study elicited instantaneous multisensory associations.

### Insula

With respect to our second alternative hypothesis proposing an involvement of frontal regions we did not find evidence for the existence of the "expectancy loo" in right dorsolateral and inferior lateral regions described by Hugdahl et al. [19]. However, we did observe robust activation in anterior insulae bilaterally in all experimental phases. Even though neuroimaging studies have so far reported involvement of the anterior insulae in a variety of sensory and cognitive tasks [49,50] the precise function of this region is still unsettled. Besides involvement in visceral sensory, visceral motor, gustatory and emotional functions the anterior insulae also appear to play a vital role in visual-audio integration and more elaborated auditory functions [51,52]. As recently pointed out there is also growing evidence which supports the view that the insula governs the detection of crossmodal coincidence [9]. From our ROI analyses we can only infer a generally stronger engagement of the right relative to the left insula regardless of the experimental phase. This observation is in agreement with a recent study that reported the right insula to support visual-auditory synchrony detection [53]. However, as this multifaceted and polysensory region appears to mediate a multitude of heterogeneous vital functions we are reluctant in providing a specific interpretation regarding the particular role the anterior insula may have played in the present study.

### Limitations of the study

First, the analyses show that the conditioning approach did not yield clear effects as we hypothesized. During the extinction phase activations in multisensory and auditory association regions were significantly weaker relative to responses to pure visual stimuli prior to conditioning. We cannot rule out that the telephone sound we used as UCS had insufficient power to form a robust and stable conditioned response. The objection may be raised that most studies of associative learning or classical conditioning use aversive stimuli as UCSs (e.g. electric shock, air puff to the cornea etc.) to achieve proper conditioned responses. Therefore, it might well have been the case that the use of aversively loud sounds would have triggered conditioning in the way we predicted.

Furthermore, we cannot be sure that responses in multisensory and auditory cortex to visual stimuli prior to the paired presentation of CS and UCS may reflect associative learning. More conclusive evidence for the interpretation of our major finding could have been achieved by the use of an autonomic measure (e.g. skin conductance response), independent from fMRI. Future studies designed to further explore this issue should therefore use autonomic measurements to complement neuroimaging results.

A further potentially limiting factor might have been that we only analysed the second out of three subsequent volumes we acquired for each trial. As the consecutive signals cannot be taken as independent events we were confronted with the issue of unsteady magnetization due to T1 decay that may systematically affect the data. Thus, we analysed the fMRI time-series for each single time point of acquisition separately and compared the outcome. The results of these separate analyses did not differ notably, therefore we present the data of the second acquisition as they are supposed to reflect the amplitude peak of the hemodynamic response.

Finally, mention should be made of one alternative interpretation which might account for our major finding of responses in the multisensory and auditory association cortex to visual stimuli in the first run. Since we applied an event-related sparse temporal acquisition approach we cannot completely rule out the possibility that participants instantly established an association between visual stimuli and scanner noise which consistently followed 3–5 s after presentation of flashes in each trial. Perhaps, volunteers learned to anticipate the onset of scanner noise each time they experienced visual stimuli followed by an auditory event. However, the present data does not allow us to judge whether the auditory activation we observed during both the first and the last run emanated from conditioning or should be considered a reflection of auditory imagery triggered by the anticipation of the scanner noise. Should the latter interpretation hold true the present finding would strongly point to a fatal side-effect of sparse temporal scanning to which researchers using this approach should be aware of. However, the observation that during phases with auditory stimulation no salient responses in multisensory and auditory association cortex were found speaks again the latter interpretation.

### General remarks

Taken together, the current data clearly show that purely visual activation could lead to an activation within multisensory and auditory association areas with the right cortical fields unveiling enhanced activation strength. We assume that the particular design and materials used in the context of this study account for this finding as we only presented nonspeech stimuli which may explain why left hemisphere regions exhibit only minor involvement.

Our present results buttresses former research showing that perceptual learning appears to occur quite automatically [54] and involves mutual interactions among multisensory brain regions associating specific sensory information with stored representations. Accordingly, Murray and colleagues demonstrated that picture presentation paired with sounds results in improved memory performance [55]. These multisensory memory representations are established extremely rapidly even after single-trial exposure and are later accessible to facilitate memory, implying an extremely fast and robust establishment of multisensory representations [56]. Even though we are not able to say whether multisensory integration takes place early in the unisensory world or later at higher stages of processing, recently published data strongly indicates that visual input speeds up cortical processing of auditory signals at an early stage [57,58] or vice versa [16]. Presently, there is mounting evidence suggesting that multisensory integration is more prevalent than previously recognized and could be considered a selective advantage in evolutionary terms. As recently outlined by Foxe and coworkers [10, p. 543]"the early detection and localization of moving and perhaps threatening objects, has clear implications for survival and the presence of coincident sensory inputs is well known to improve detection and localization". Based on our finding we reason that a purely visual stimulus elicited responses which recruit neural ensembles in multisensory and auditory associative cortices. In other words, we assume that the perisylvian and STS activation we observed should be considered part of a crossmodal network which is responsive to simple sensory information to enable rapid associative learning. Advanced methodological approaches like "silent" fMRI and MR machines with such a high field strength as the one used in the present study providing improved spatial resolution may account for the fact that insights not envisaged a decade ago are now being gained.

## 4 Conclusion

In the present event-related sparse temporal fMRI study we paired a visual stimulus (doubled red flash) with an auditory stimulus (ringing of a telephone). In the absence of auditory stimulation the presentation of visual stimulation elicited bilateral, but right dominant activation in the auditory association and heteromodal cortex (posterior Sylvian fissure, posterior STS). We observed auditory activation evoked by previously unrelated visual stimuli without instructing the participants to explicitly imagine the sounds of responses prior to and following the paired audiovisual presentation. Thus, the present study demonstrates general and instantaneous involvement of heteromodal and auditory association areas in perception of unimodal visual stimulation which may reflect the forming of multisensory associations that cannot be attributed to *sensation *of an auditory event. Apparently the visual stimuli (CS) used in this study were not affectively neutral as it was originally intended but due to its apparent behavioural relevance provoked rapid association between visual events and auditory or somatosensory representations. The question of whether this interpretation holds true or whether participants build up a triggered relationship between visual events and subsequent scanner noise emitted by acquisition of three single fMRI volumes reflecting an anticipatory process requires further, more refined studies utilizing auditory stimuli as CS and the application of autonomic measurements, e.g. skin conductance responses that measure excitement independent from fMRI.

## 5 Methods

### Participants

Sixteen healthy volunteers (8 males, 8 females, age range 24–40, mean 27.8 yrs.), all strongly right-handed according to a standard questionnaire [59,60], partook in the study. Volunteers were not familiarized with the stimuli or procedure prior to scanning. They had no neurological or psychiatric illness, nor did they have any visual or hearing disorder. Written informed consent was obtained prior to the examination. The study was approved by the local Ethical Committee of Zurich Medical Faculty. Due to motion artefacts one participant had to be excluded from analysis.

### Experimental setup and stimuli

**Figure 3:**
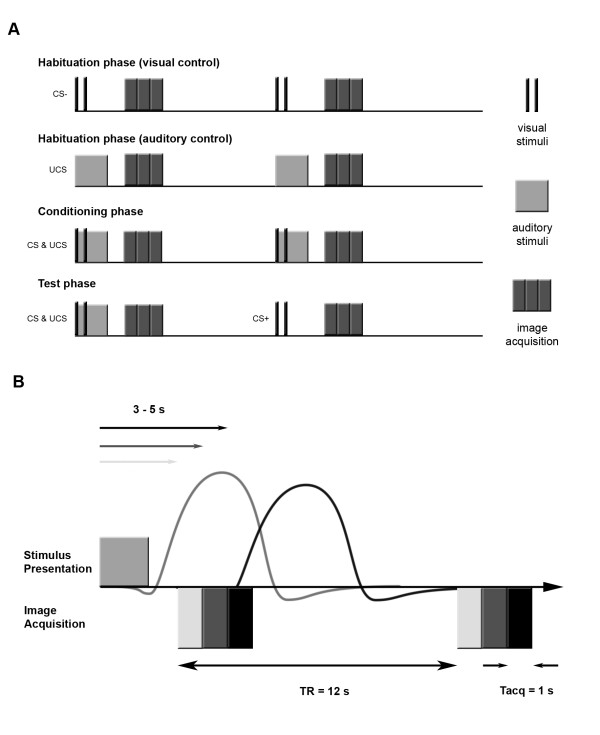
[A] Schematic illustration of the four experimental phases. For details see the Methods section. [B] Schematic illustration of clustered temporal acquisition (CTA) as implemented in the present study. In each single trial the hemodynamic response is obtained from three consecutive volume scans (Tacq 1 s). The interval between onset of one volume triplet and onset of the subsequent triplet is 12 s. The interval between onset of stimulus presentation and data collection varies between 3 and 5 sec and allows enhanced sampling of data points relative to single volume acquisition. This approach precludes confounding hemodynamic responses to stimuli with scanner noise.

The study comprised a visual and an auditory stimulus. The visual stimulus was either presented in isolation or paired with the auditory stimuli (Figure 3A, first and fourth row). We used a total screen red flash which lighted up for 100 ms followed by a total dark screen (100 ms) which was again replaced by a red flash (100 ms). A telephone ringing (MP3 download) [61] served as auditory stimulus and was either presented in isolation or paired with the visual stimuli (Figure 3A, second, third, and fourth row). The sound signal was digitised at a 16 bit/44.1 kHz sampling rate and shortened to 2.6 s using the Magix Deluxe software [62]. Stimuli were controlled using Presentation^© ^software [63]. Stimulus presentation was synchronized by a 5 V TTL trigger pulse with the data acquisition. We used standard Phillips headphones for binaural stimulus delivery. Null events that were randomly interspersed and during which neither auditory nor visual stimuli were presented served as silent control for data analysis. During null events participants viewed a black screen throughout the entire trial.

### Experimental procedure and task

Prior to scanning participants were informed about the experimental procedure but not about the scientific background of the study. Volunteers' task was to attend to the stimuli and to press a button alternately with the right and left index finger after each trial signalled by the offset of scanner noise. As associative learning is supposed to occur automatically we had our participants perform this simple task, specifically not directing the subjects' attention to the stimuli, but to the scanner noise. The task was designed to keep participants generally attentive. Participants were comfortably placed supine in the scanner and underwent four experimental blocks. Each block corresponded to one particular experimental phase that we introduce in turn.

The first visual phase served as a visual control condition as participants only viewed visual stimuli in isolation (CS, n = 32) and randomly interspersed null events (n = 16). The second habituation phase served as an auditory control condition since volunteers only heard auditory stimuli in isolation (UCS, n = 32) and randomly interspersed null events (n = 16). During the third phase (conditioning) we consistently presented paired visual and auditory stimuli (CS and UCS, n = 32) and randomly interspersed null events (n = 16). During the fourth phase participants were either presented with paired visual and auditory stimuli (CS- and UCS, n = 32) as in the preceding phase, visual stimuli in isolation (CS+, n = 32), or randomly interspersed null events (n = 32). In other words, we applied a 5:10 reinforcement plan to partly maintain conditioning and to preclude fast extinction of the established association. While the duration of first, second, and third block was 12 minutes each, the scanning of the last phase took 24 minutes resulting in a total of 60 minutes scanning time for the functional part of the experiment. All participants experienced the same order of experimental phases. Generally, the sequence of visual, auditory, and null events was pseudo-randomised within each block to preclude predictability.

### Experimental design

To avoid a perceptual and physiological masking of auditory processing induced by scanner noise we applied a "silent" fMRI protocol (clustered-sparse temporal acquisition scheme, CTA). This approach combines the principle design of a sparse temporal acquisition (STA) with the clustered acquisition of three consecutive volume scans per trial [64]. A long inter-scan interval (repetition time 15 s) then allows both the functional response to the auditory stimulus and the response evoked by the scanner noise to decay prior to the next trial (see Figure 3B). This approach is capable of clearly separating the task-induced functional response from the scanner-noise induced functional response.

### Data acquisition

Data were collected using a Philips Intera 3 T whole body MR unit (Philips Medical Systems, Best, The Netherlands) equipped with an eight-channel Philips SENSE head coil. Functional time series were obtained from 14 transverse slices covering the entire perisyl-vian cortex with a spatial resolution of 2.7 × 2.7 × 4 mm using a Sensitivity Encoded (SENSE; [65]) single-shot gradient-echo planar sequence (acquisition matrix 80 × 80, SENSE acceleration factor R 2.0, FOV 220 mm, TR 1000 ms, TE 35 ms and flip angle 90°). Additionally, we obtained one echo planar image that covered the whole brain with 38 transverse slices (TR 4000 ms) but applied otherwise the identical scan parameters as used with the functional time series. This whole-head EPI volume was used to assist the spatial normalization of the functional time series (c.f. Data Analysis). Furthermore, we collected a standard 3D T1 weighted scan for anatomical reference with 1 × 1 × 0.8 mm spatial resolution (acquisition matrix 224 × 224, TE 2.30 ms, TR 20 ms, flip angle 20°).

### Data analysis

To account for different T1 saturation effects in subsequent volumes, we subjected the three volume scans collected during each cluster to three separate time series during data analysis. Each of these three time-series corresponded to the hemodynamic response sampled at a distinct temporal window, i.e. 3 s, 4 s and 5 s after stimulus onset.

Pre- and post-processing of fMRI time-series were carried out using MATLAB 6.5 (Mathworks Inc., Natiek, MA, USA) and the SPM99 software package [66]. All volumes were realigned to the first volume, corrected for motion artefacts, mean-adjusted by proportional scaling, normalized into standard stereotactic space [67]. In order to optimise normalization we coregistered the functional time-series with the whole-head EPI-T1 images. For spatial smoothing we applied an isotropic Gaussian kernel (8 mm full-width-at-half-maximum). Low-frequency drifts were removed using a temporal high-pass filter (cut-off of 100 s).

Statistical analysis was based on the General Linear Model [68]. Single trials were treated as epochs and modelled by means of a box car function. We calculated contrast images from each of the three volumes. The resulting set of voxel values for each contrast constitutes a statistical parametric map of the T-statistic [SPM(*T*)]. In order to explore the group-level activation across the 15 participants we used a random effects model (second level analysis on contrast images obtained from individuals). This model estimates the error variance for each phase across individual subjects rather than across all scans and thus provides stronger generalization of the statistical population. Due to unsteady magnetization associated with the clustered temporal acquisition we only report activity collected with the second out of the three clustered trials. For report and discussion of results only significant clusters of activation were considered (uncorrected *α*-level 0.001, *k *≥ 10).

We also performed a post hoc 'region of interest' (ROI) analysis which enabled us to test whether BOLD responses obtained from distinct sites of the fronto-temporo-parietal cortex may vary as a function of phase. For four conditions (visual habituation, auditory habituation, paired audiovisual presentation, and extinction) we collected BOLD signals recorded during the second out of three volumes from five bilateral ROIs placed in the anterior insula, in the mid portion of the STG, in the planum temporale, in the posterior superior temporal sulcus (STS), and in the supramarginal gyrus (SMG) overarching the planum parietale (PPa) from all participants. Spherical ROIs (radius 4 mm) were defined as this approach guarantees homogeneity of variance due to the equal size of ROIs [69]. We defined coordinates of averaged local response maxima as centre voxels of ROIs (cf. Tables 1, 2, 3, 4): LH STR/HG (-40, -28, 7), RH STR/HG (49, -18, 5), LH SMG/PPa (-60, -21, 27), RH SMG/PPa (63, -36, 27), LH PT (-62, -38, 18), RH PT (62, -38, 18), LH insula (-30, 22, 3), RH insula (34, 21, -3), LH STS (-60, -42, -3), RH STS (60, -42, -3). *β*-values were averaged within each distinct spherical ROI, across experimental phases, participants and hemispheres and subjected to systematic ANOVAs.

## Authors' contributions

MM designed the experimental paradigm, performed the ROI analysis and drafted the manuscript. SB built the experimental setup, programmed the experimental stimulation and scanning, performed the postprocessing of the data and contributed to the manuscript. SM conducted the fMRI scanning and performed the preprocessing of the data. LJ contributed to the hypothesis, design, results, discussion, and to the preparation of the manuscript.

Herewith the corresponding authors confirms that all authors read and approved the final manuscript.

## Figures and Tables

**Table 1:** Vision vs. null events

*Vision *> *Silent Control*	Left hemisphere					Right hemisphere				
Location	k	T value	x	y	z	k	T value	x	y	z

*CLS/LG/Cun*	511	4.93	0	-93	3	-	-	-	-	-
*MTG/MOG*	36	4.96	-54	-69	3	-	-	-	-	-
*SMG/PPa*^*a*^	162	4.47	-60	-21	27	-	4.69	63	-36	27
*STG/PT*^*a*,*b*^	-	-	-	-	-	-	4.46	63	-36	15
*STS*^*c*^	-	-	-	-	-	263	5.17	60	-42	-3
*Ins/TP*	197	4.46	-42	15	-6	184	4.67	57	9	-6
*Ins*	-	-	-	-	-	d	4.23	40	24	-12
*IFG(op)*	-	-	-	-	-	28	4.52	60	15	12
*Put*	24	4.13	-24	12	-9	-	-	-	-	-
*Tha*	10	3.90	-3	-12	9	-	-	-	-	-

^*a *^This cluster cannot be distinctively separated by the cluster in the right STG. Thus this cluster has a local maximum, but no distinct extension.

^*b*^According to the probability atlas by Westbury et al. [70] the local maximum of this cluster is situated in the PT with a 5–25% probability.

^*c*^According to the probability atlas by Westbury et al. [70] the local maximum of this cluster is situated outside the PT.

^*d *^This cluster cannot be distinctively separated by the cluster in the adjacent TP. Thus this cluster has a local maximum, but no distinct extension.

Local response maxima of significant clusters (random-effects analysis, *p *≤ 0.001 uncorrected for multiple comparisons; *k *≥ 10). Localization of clusters correspond to position of local maximal activations indicated by the T value in normalized space of the Montreal Neurological Institute (MNI) standard brain [67] for a particular anatomical structure. Distances are relative to the intercommissural (AC-PC) line in the horizontal (*x*), anterior-posterior (*y*) and vertical (*z*) directions. We used the "Automatic anatomical labelling" tool [71,72] available for implementation in SPM99 [66]. Anatomical abbreviations are defined as follows: IFG(op) = inferior frontal gyrus (opercular part), IFG(tr) = inferior frontal gyrus (triangular part), HG = Heschl's gyrus, STG = superior temporal gyrus, STS = superior temporal sulcus, PT = planum temporale, PPa = planum parietale, MTG = middle temporal gyrus, SMG = supramarginal gyrus, TP = temporal pole, MOG = middle occipital gyrus, LG = lingual gyrus, CLS = calcarine sulcus, Cun = Cuneus, PCun = Precuneus, Ins = Insula, Tha = Thalamus, IC = inferior colliculus.

**Table 2:** Audition vs. null events.

*Audition *> *Silent Control*	Left hemisphere					Right hemisphere				
Location	k	T value	x	y	z	k	T value	x	y	z

*STG*^*a*^	789	6.62	-36	-30	9	1003	6.73	48	-18	6
*Ins*	24	3.92	-30	24	3	24	4.08	36	21	-3
*Tha*	26	4.40	-9	-9	6	33	3.92	9	-18	3

^*a *^The cluster in the right hemisphere also encompassed the Rolandic operculum. According to the probability atlas by Rademacher et al. [73] the local maximum of this cluster is situated in the HG with 40% probability.

Local response maxima of significant clusters (random-effects analysis, *p *≤ 0.001 uncorrected for multiple comparisons; *k *≥ 10). For explanations, see Table 1.

**Table 3:** Paired vision (CS) and audition (UCS) vs. null events.

*CS and UCS *> *Silent Control*	Left hemisphere					Right hemisphere				
Location	k	T value	x	y	z	k	T value	x	y	z

*STG/Ins*	600	5.68	-45	-27	6	857	6.54	51	-18	3
*CLS*^*a*^	274	4.42	-15	-75	9	-	-	-	-	-
*LG*	14	3.72	-21	-57	0	-	-	-	-	-
*Cun*^*a*^	-	-	-	-	-	90	4.37	9	-75	39
*IC*	-	-	-	-	-	15	4.14	9	-24	-9

^*a *^Activation occurs in both left and right hemisphere with only the maximum T-value peaking in the indicated hemisphere.

Local response maxima of significant clusters (random-effects analysis, *p *≤ 0.001 uncorrected for multiple comparisons; *k *≥ 10). For explanations, see Table 1.

**Table 4:** Vision (CS+) vs. null events.

*CS+ *> *Silent Control*	Left hemisphere					Right hemisphere				
Location	k	T value	x	y	z	k	T value	x	y	z

*STG/PT*^*a*^	-	-	-	-	-	84	4.45	60	-39	21
*IFG(tri)*	-	-	-	-	-	27	4.09	48	12	27
*Ins*	98	5.26	-30	21	3	14	3.74	33	21	0
*Ins/TP*	-	-	-	-	-	31	4.08	51	9	-9
*CLS*^*b*^	214	4.30	-15	-72	6	-	-	-	-	-
*LG*	-	-	-	-	-	16	4.37	21	-51	-6
*PCun/Cun*	-	-	-	-	-	20	3.69	9	-75	39
*Tha*	24	3.90	-9	-18	6	-	-	-	-	-
*IC*	16	4.63	-9	-27	-9	-	-	-	-	-

^*a*^According to the probability atlas by Westbury et al. [70] the local maximum of this cluster is situated in the PT with 26–65% probability.

^*b *^Activation occurs in both left and right hemisphere with only the maximum T-value peaking in the indicated hemisphere.

Local response maxima of significant clusters (random-effects analysis, *p *≤ 0.001 uncorrected for multiple comparisons; *k *≥ 10). For explanations, see Table 1.

**Table 5:** Activation within regions of interest.

**Region**	**Factor**	**DF**	**F Value**	**P Value**
**anterior insula**	*Phase*	3,11	2.37	ns
	*Hemi*	1,14	6.3	*p *< .05
	*Phase *× *Hemi*	3,11	3.4	*p *< .05
**planum parietale/supramarginal gyrus**	*Phase*	3,11	2.58	ns
	*Hemi*	1,14	1.09	ns
	*Phase *× *Hemi*	3,11	3.77	*p *< .05
**Heschl's gyrus/STG**	*Phase*	3,11	99.25	*p *< .0001
	*Hemi*	1,14	23.59	*p *< .0001
	*Phase *× *Hemi*	3,11	147.26	*p *< .0001
**planum temporale**	*Phase*	3,11	2.57	ns
	*Hemi*	1,14	0.58	ns
	*Phase *× *Hemi*	3,11	1.8	ns
**posterior superior temporal sulcus**	*Phase*	3,11	4.373	*p *< .05
	*Hemi*	1,14	36.85	*p *< .0001
	*Phase *× *Hemi*	3,11	1.48	ns

Results of a (2 × 2) ANOVA based on *β*-values with factors *hemisphere *× *phase *within each distinct ROI.
